# Leucine-Rich α-2-Glycoprotein 1 Suppresses Endothelial Cell Activation Through ADAM10-Mediated Shedding of TNF-α Receptor

**DOI:** 10.3389/fcell.2021.706143

**Published:** 2021-07-05

**Authors:** Kuin Tian Pang, Mean Ghim, Chenghao Liu, Hui Min Tay, Chee Wai Fhu, Rui Ning Chia, Beiying Qiu, Padmini Sarathchandra, Adrian H. Chester, Magdi H. Yacoub, Fiona L. Wilkinson, Ria Weston, Christina M. Warboys, Han Wei Hou, Peter D. Weinberg, Xiaomeng Wang

**Affiliations:** ^1^Department of Bioengineering, Imperial College London, London, United Kingdom; ^2^Institute of Molecular and Cell Biology, Agency for Science, Technology and Research (A^∗^STAR), Singapore, Singapore; ^3^Lee Kong Chian School of Medicine, Nanyang Technological University, Singapore, Singapore; ^4^School of Mechanical and Aerospace Engineering, Nanyang Technological University, Singapore, Singapore; ^5^Cancer Science Institute of Singapore, National University of Singapore, Singapore; ^6^Centre for Vision Research, Duke-NUS Medical School, Singapore, Singapore; ^7^Harefield Heart Science Centre, National Heart and Lung Institute, Imperial College London, London, United Kingdom; ^8^Department of Life Sciences, Manchester Metropolitan University, Manchester, United Kingdom; ^9^Comparative Biomedical Sciences, The Royal Veterinary College, London, United Kingdom; ^10^Singapore Eye Research Institute, Singapore, Singapore

**Keywords:** leucine-rich α-2-glycoprotein 1, NF-κB, TNFR1 shedding, endothelial activation, coronary artery disease, critical limb ischemia, atherosclerosis, flow

## Abstract

Elevated serum concentrations of leucine-rich α-2-glycoprotein (LRG1) have been reported in patients with inflammatory, autoimmune, and cardiovascular diseases. This study aims to investigate the role of LRG1 in endothelial activation. LRG1 in endothelial cells (ECs) of arteries and serum of patients with critical limb ischemia (CLI) was assessed by immunohistochemistry and ELISA, respectively. LRG1 expression in sheared and tumor necrosis factor-α (TNF-α)-treated ECs was analyzed. The mechanistic role of LRG1 in endothelial activation was studied *in vitro*. Plasma of 37-week-old *Lrg1*^–/–^ mice was used to investigate causality between LRG1 and tumor necrosis factor receptor 1 (TNFR1) shedding. LRG1 was highly expressed in ECs of stenotic but not normal arteries. LRG1 concentrations in serum of patients with CLI were elevated compared to healthy controls. LRG1 expression was shear dependent. It could be induced by TNF-α, and the induction of its expression was mediated by NF-κB activation. LRG1 inhibited TNF-α-induced activation of NF-κB signaling, expression of VCAM-1 and ICAM-1, and monocyte capture, firm adhesion, and transendothelial migration. Mechanistically, LRG1 exerted its function by causing the shedding of TNFR1 via the ALK5-SMAD2 pathway and the subsequent activation of ADAM10. Consistent with this mechanism, LRG1 and sTNFR1 concentrations were correlated in the serum of CLI patients. Causality between LRG1 and TNFR1 shedding was established by showing that *Lrg1*^–/–^ *mice* had lower plasma sTNFR1 concentrations than wild type mice. Our results demonstrate a novel role for LRG1 in endothelial activation and its potential therapeutic role in inflammatory diseases should be investigated further.

## Introduction

Endothelial cells (ECs) line the inner surface of all blood vessels and are important in maintaining vascular homeostasis. In the physiological state, ECs tightly regulate vascular tone, vessel permeability, and leukocyte adhesion (Godo and Shimokawa, 2017). Vascular homeostasis breaks down when the endothelium is activated, which can be triggered by factors such as pro-inflammatory cytokines, hyperglycemia, and pro-atherogenic shear stress (Beckman et al., 2002; Sprague and Khalil, 2009; Wang et al., 2013a; Ghim et al., 2018). Such dysfunction of the endothelium is critical in the development of atherosclerosis, a chronic inflammatory disease that involves excessive net uptake of low density lipoprotein (LDL) (Zhang et al., 2014; Ghim et al., 2017; Mundi et al., 2018) and monocyte recruitment (Mestas and Ley, 2008; Ghim et al., 2018).

The nuclear factor kappa B (NF-κB) pathway is central to the breakdown and resolution of endothelial homeostasis (Pober, 2002). Binding of tumor necrosis factor-α (TNF-α) to tumor necrosis factor receptor 1 (TNFR1) activates the NF-κB signaling cascade, which requires phosphorylation of protein kinase B (Akt) (Nidai Ozes et al., 1999). NF-κB activation also involves inhibitor kappa B alpha (IκBα) phosphorylation, leading to the translocation of NF-κB from the cytosol to the nucleus (Hoffmann et al., 2002) and the subsequent transcription of endothelial adhesion molecules such as vascular cellular adhesion molecule-1 (VCAM-1) and intercellular adhesion molecule-1 (ICAM-1) and intercellular adhesion molecule-1 (ICAM-1) (Pober, 2002; van der Heiden et al., 2010). Binding of TNF-α to TNFR1 also induces the activation of apoptotic signaling by releasing an active form of caspase-8 (Wang et al., 2008), which in turn cleaves and activates caspase-3 (Enari et al., 1998). Cleaved caspase-3 then targets a latent DNAse that degrades chromosomal DNA (Sedger and McDermott, 2014).

Although knocking out components of the NF-κB pathway generally protects mice from the development of atherosclerosis (Bourdillon et al., 2000; Cybulsky et al., 2001; Bråneìn et al., 2004), one study showed that macrophage-specific deletion of IκB kinase 2 (IKK2) leads to increased atherosclerotic lesion formation and an impaired inflammatory response (Kanters et al., 2003). NF-κB activation has also been shown to play a role in the resolution of inflammation by inducing the expression of anti-inflammatory genes and apoptosis (Lawrence et al., 2001). Therefore, a better understanding of the NF-κB pathway is required to facilitate the development of NF-κB-targeted therapeutics for the treatment of atherosclerosis.

Leucine-rich alpha-2-glycoprotein 1 (LRG1) is a novel regulator of angiogenesis and it exerts its function by activating the transforming growth factor-β (TGF-β) signaling pathway in ECs (Wang et al., 2013b). Besides its role in angiogenesis, LRG1 is intimately linked to tissue inflammation. TNF-α has been shown to induce LRG1 expression in ECs (Wang et al., 2017) and elevated LRG1 expression was observed during granulocyte differentiation (O’Donnell et al., 2002). Furthermore, elevated serum LRG1 levels were reported in human patients with various inflammatory, autoimmune, and cardiovascular diseases (Watson et al., 2011; Serada et al., 2012; Ha et al., 2014; Yin et al., 2014; Pek et al., 2015; Bos et al., 2017; Xie et al., 2019). However, its role in endothelial activation and atherosclerosis remains to be elucidated.

In the present study, we showed that LRG1 expression is induced in activated ECs and the endothelium of stenosed coronary arteries. As atherosclerosis is the principal cause of most critical limb ischemia (CLI), we evaluated circulating LRG1 levels in CLI patients and demonstrated a positive correlation between LRG1 and soluble TNFR1 in CLI. Surprisingly, LRG1 inhibited NF-κB signaling, the expression of VCAM-1 and ICAM-1, and monocyte capture, firm adhesion, and transendothelial migration. Mechanistically, LRG1 exerted its function by inducing the shedding of TNFR1 via the ALK5-SMAD2 pathway as well as the activation of ADAM10. Together, these findings establish a novel role for LRG1 in endothelial activation and suggest that LRG1 may have a significant atheroprotective effect.

## Materials and Methods

### Patient Samples

Five-μm-thick sections of formalin-fixed, paraffin-embedded left anterior descending epicardial coronary arteries that were free of stenosis (*n* = 3) or stenosed (*n* = 6) were obtained from heart transplant recipients at Harefield Hospital, United Kingdom. The samples originated from a historical biobank of specimens collected prior to September 2006 and the implementation of the Human Tissue Act in the United Kingdom. Tissues were collected and stored with the permission of the patients who at the time of their transplant had expressed no objection to tissue from their explanted heart being used for research and teaching purposes.

Peripheral blood samples were collected from patients with CLI with or without diabetes on the day before their lower limb amputation, or healthy volunteers (*n* = 11/group). Patient details are given in Supplementary Table 1A. Blood samples were centrifuged to collect serum for analysis.

All human blood was obtained with informed consent and procedures were in accordance with institutional guidelines and the Declaration of Helsinki (Ethical reference: 14/NW/1062, approved by Manchester Metropolitan University Internal Ethics Approval Committee).

### Elastic Van Gieson (EVG) Staining of Coronary Artery Sections

After deparaffinisation and rehydration, the coronary artery sections were immersed in Miller’s elastin stain for 2 h at room temperature, followed by three washes with deionized (DI) water. After the washing steps, sections were immersed in Van Gieson stain for 5 min at room temperature to stain elastic fibers (dark purple), collagen (pink to red), and muscle (yellow). Stained sections were rinsed with DI water before being dehydrated in a graded methanol series and cleared with Histo-Clear. Slides were mounted in DPX (Sigma-Aldrich, United Kingdom).

### Immunohistochemical Staining of Coronary Artery Sections

After deparaffinisation and rehydration, antigens were retrieved by soaking sections in citrate buffer (pH 6, Sigma-Aldrich, United Kingdom) for 30 min at 100°C. After cooling and rinsing with DI water, sections were incubated with 3% hydrogen peroxide for 10 min to block endogenous peroxidase activity. Non-specific binding was blocked by incubating the sections with 10% goat serum in Tris-buffered saline (TBS) for 45 min. The sections were incubated with rabbit anti-LRG1 antibody (13224-1-AP, Proteintech, United States) or mouse anti-CD31 antibody (JC70A, Agilent, United States) at a 1:400 dilution in TBS buffer containing 0.1% bovine serum albumin (BSA) overnight at 4°C, followed by incubation with mouse anti-rabbit horseradish peroxidase-conjugated secondary antibody (sc-2357, Santa Cruz Biotechnology, United States) at a 1:200 dilution in TBS buffer containing 0.1% BSA for 1 h at room temperature. The slides were then incubated in substrate reagent containing diaminobenzidine (DAB+, Agilent, United States) for 5 min. Nuclei were counter-stained in Gill’s Haematoxylin (Sigma-Aldrich, United Kingdom) for 30 s. Slides were mounted using Canada Balsam (Sigma-Aldrich, United Kingdom) after sequential dehydration steps. The percentage of LRG1-positive ECs was calculated manually.

### Enzyme-Linked Immunosorbent Assay

The concentration of LRG1 and sTNFR1 in the serum of CLI patients and healthy controls as well as in *Lrg1*^–/–^ mice was measured using a human LRG1 ELISA (IBL, Japan) and a human or mouse sTNFR1 ELISA (R&D Systems, United States) according to the manufacturer’s protocol.

### Cell Isolation and Culture

Umbilical cords were obtained from donors with uncomplicated labor at the Hammersmith Hospital, United Kingdom. Isolation and culture of human umbilical vein endothelial cells (HUVECs) was approved for research purposes by the Hammersmith Hospital Research Ethics Committee (ref. 06/Q0406/21). HUVEC isolation was performed as described by Jaffe et al. (1973) with minor modifications. Human aortic endothelial cells (HAECs) were obtained from Lonza (Switzerland). Both cell types were cultured on 0.1% gelatin-coated flasks in Endothelial Cell Growth Medium (EGM-2) containing the EGM-2 supplement kit (Lonza, Switzerland). Human dermal microvascular endothelial cells (HDMECs) were obtained from PromoCell (Germany) and were cultured in Microvascular Endothelial Cell Growth Medium-2 (EGM-2 MV) containing the EGM-2 MV supplement kit (Lonza, Switzerland). Cells between passages 3–5 were used for experiments. HUVECs were used for TNF-α and shear application, transfection, signaling studies, the dose-response study of recombinant human LRG1 (rhLRG1) in monocyte adhesion, and the monocyte capture assay. HAECs were used for TNF-α application, monocyte-endothelial interaction assays and TNFR1 shedding studies. HDMEC were used for chromatin immunoprecipitation (ChIP) assay. Human acute monocytic leukemia suspension line (THP-1) cells (ATCC, United States) were maintained in RPMI 1640 medium supplemented with 10% Fetal Bovine Serum (FBS), 2 mM L-glutamine, 100 U/mL penicillin and 100 μg/mL streptomycin (all from Sigma-Aldrich, United Kingdom). Passages 6–20 were used for experiments. All three cell lines were maintained at 37°C in a humidified incubator under 95% air/5% CO_2_.

Freestyle human embryonic kidney 293 cells (HEK293F, Life Technologies, United Kingdom) were maintained in Freestyle 293F Expression Medium (Life Technologies, United Kingdom) on an orbital shaker (130 rpm) at 37°C in a humidified incubator under 92% air/8% CO_2_.

### Application of Shear Stress

Endothelial cells grown at the center and the edge of a six-well plate experience, respectively, low magnitude multidirectional flow (LMMF) (putatively atherogenic) and high magnitude uniaxial flow (HMUF) (putatively atheroprotective) when the wells are swirled on the horizontal platform of an orbital shaker (Ghim et al., 2018). To ensure that cell seeding was restricted to either the center or the edge, the wells were coated with fibronectin (Sigma-Aldrich, United Kingdom) in one region, followed by passivation of the other region with Pluronic F-127 (Sigma-Aldrich, United Kingdom) (Ghim et al., 2018; Pang et al., 2021). After seeding, ECs were allowed to grow to confluence before being placed on the orbital shaker (POS-300, Grant Instruments) inside the humidified incubator for another 3 days. The platform orbited in the horizontal plane with an orbital radius of 5 mm and angular velocity of 150 rpm.

### SDS-PAGE and Western Blotting

Endothelial cells were lysed using radioimmunoprecipitation assay (RIPA) buffer (Sigma-Aldrich, United Kingdom) supplemented with Halt protease and phosphatase inhibitor (Thermo Fisher Scientific, United States). Extracted proteins were separated by sodium dodecyl sulfate-polyacrylamide gel electrophoresis (SDS-PAGE) before being transferred onto a polyvinylidene fluoride (PVDF) membrane (Merck Millipore, United States). Blots were incubated with primary antibodies overnight at 4°C, followed by horseradish peroxidase-conjugated secondary antibodies for 1 h at room temperature. (Antibodies and dilutions are given in Supplementary Table 2) Glyceraldehyde 3-phosphate dehydrogenase (GAPDH) and calnexin were used as housekeeping genes for static and shear experiments, respectively. Blots were incubated with Clarity ECL substrate (Bio-Rad, United States) before being imaged using a Biospectrum imaging system (UVP, United Kingdom). Densitometry was performed using Image Studio Lite software (LI-COR, United States).

### Chromatin Immunoprecipitation (ChIP)

Human dermal microvascular endothelial cells were cross-linked with 1% formaldehyde (Sigma-Aldrich, United Kingdom) for 10 min before being quenched with 0.125 M glycine for 5 min. Cells were then lysed in SDS lysis buffer containing 1% SDS, 5 mM ethylenediaminetetraacetic acid (EDTA), 50 mM Tris–HCL (pH 8.1) and 1× complete, mini, EDTA-free protease inhibitor cocktail (Roche, United States) for 10 min on ice. Cell lysates were sonicated into 500 bp fragments with a Vibra-Cell processor (Sonics, United States). Sheared chromatin was cleared of debris and incubated overnight at 4°C with either rabbit IgG (Sigma-Aldrich, United Kingdom), or 2 μg rabbit polyclonal anti-NF-κB p65 antibody (Santa Cruz, United States). After washing, the protein-DNA crosslinks were reversed and DNA was eluted in 240 μL elution buffer containing 1% SDS and 0.1 M sodium bicarbonate (NaHCO_3_). DNA purification was performed using the QIAquick PCR purification kit (Qiagen, Germany) according to the manufacturer’s instructions. qRT-PCR was performed using the primers spanning two NF-κB binding sites near the LRG1 transcription start site (TSS). Binding sites located in fragments (−2000 to +2000 from TSS) that interact with LRG1 gene were predicted by motif analysis using FIMO (Grant et al., 2011).

Region 1, forward primer, 5′-CCAGGAATAGTGCCTTGC AAA-3′, reverse primer, 5′-GCCTTATACCTGCCTGGAC TGG-3′.
Region 2, forward primer, 5′-GCACACACACACACACCC CTA-3′, reverse primer, 5′-GCTCACTGCAGCCTCTGAA-3′

### Overexpression of LRG1 in ECs

Leucine-rich α-2-glycoprotein (LRG1) was overexpressed by transfecting pcDNA-LRG1-HIS plasmid into HUVECs using Lipofectamine 3000 (Thermo Fisher Scientific, United Kingdom). The pcDNA-LRG1-HIS plasmid was constructed as previously described (Wang et al., 2013b). Empty pcDNA3.1 plasmid was used as a control plasmid.

### Production of Recombinant LRG1 Protein

The LRG1 plasmid was transfected into HEK293F cells using Lipofectamine 3000 according to the manufacturer’s protocol. G418 (Thermo Fisher Scientific, United States) was used to select a stable LRG1-overexpressing HEK293F cell line. Conditioned medium was concentrated using Amicon Ultra-15 Centrifugal Units (Merck Millipore, United States). rhLRG1 was purified using Ni Sepharose beads (GE Healthcare, United States) overnight at 4°C on a roller. Eluted LRG1 was buffer-exchanged into phosphate-buffered saline (PBS) using Amicon Ultra-15 Centrifugal Units. The concentration of rhLRG1 was measured by Bradford protein assay (Bio-Rad, United States).

### Recombinant Protein and Inhibitor Treatment

To investigate the effect of LRG1 on endothelial activation, ECs were pre-treated with rhLRG1 (250 μg/mL unless otherwise stated) for 24 h prior to activation with TNF-α (10 ng/mL, PeproTech, United Kingdom) for another 24 h. PBS was used as a vehicle control.

To investigate the mechanism of the effect of LRG1 on TNFR1 shedding, ECs were pre-treated with inhibitors of ADAM10 (GI254023X, 10 μM; Sigma-Aldrich, United Kingdom), ALK1 (LDN193189, 100 nM; Sigma-Aldrich, United Kingdom), or ALK5 (SB431542, 10 μM; Stratech Scientific, United Kingdom) for 1 h, followed by the treatment with 250 μg/mL of rhLRG1 for another 23 h. PBS and/or Dimethyl Sulfoxide (DMSO) were used as vehicle controls. ECs were cultured in Endothelial Cell Growth Basal Medium-2 (EBM-2, Lonza, Switzerland) supplemented with 0.2% FBS for inhibitor studies.

### THP-1 Capture Assay

Twenty μL of HUVECs at a concentration of 1.5 × 10^6^/mL were seeded into a microfluidic device with a straight channel (400 μm width × 100 μm height) and were grown to confluence in a humidified incubator. The cells were treated with 10 ng/mL TNF-α for 24 h after pre-treatment with rhLRG1 or vehicle control for 24 h (250 μg/mL). The device was then mounted on a microscope stage-top incubator maintained at 37°C and the channel was washed twice with PBS. Using a peristaltic pump (P720, Instech Laboratories), THP-1 at a density of 1.5 million cells per mL were perfused for 15 min over the HUVEC monolayer at a rate that gave a shear stress of 1 dyne/cm^2^. The channel was subsequently washed with RPMI 1640 medium for another 10 min to remove unbound cells. Attached cells were visualized under a microscope and counted manually. The setup of the assay is shown in Figure 3B.

### THP-1 Adhesion Assay

One μL of calcein-AM solution (1 mg/mL, Life Technologies, United States) was added to 1 mL of the THP-1 cell suspension at 1 million cells per mL in RPMI 1640 medium and incubated for 30 min in a humidified incubator. THP-1 cells were then centrifuged at 200 x g before being resuspended in EGM-2 medium. One million THP-1 cells were applied to a monolayer of ECs seeded in a six-well plate for 1 h in a humidified incubator, followed by three washes with pre-warmed RPMI medium to remove unbound THP-1. The EC had been maintained under static conditions or swirled on the orbital shaker prior to the assay whereas the THP-1 cells were added and incubated under static conditions in both cases.

For ECs that had been cultured under static conditions, fluorescence from adhered THP-1 cells was measured using a plate reader (SpectraMax M5, Molecular Devices) with excitation and emission wavelengths of 495/520 nm. Relative fluorescence units (RFUs) were converted to the absolute number of adhered THP-1 using a standard curve.

For ECs that had been cultured under shear, adhered THP-1 were fixed with 4% PFA for 15 min. Ten random fields were imaged using an inverted fluorescence microscope (SP105F, Brunel Microscopes) with a 20× objective, 470/40 nm excitation filter, 495 nm dichroic mirror and 525/50 nm emission filter. The number of adhered THP-1 cells was quantified using a custom MATLAB script. The number of adhered THP-1 was normalized by the number of HAECs (Ghim et al., 2018).

### THP-1 Transmigration Assay

Endothelial cells were seeded into a 6.5 mm-diameter Transwell^®^ with 8 μm pore membrane and grown to confluence. Two hundred thousand Calcein AM-stained THP-1 were resuspended in basal RPMI 1640 medium supplemented with 2% FBS before being added to the top chamber of the Transwell^®^. Monocyte chemoattractant protein-1 (MCP-1) at a concentration of 100 ng/mL in RPMI 1640 supplemented with 10% FBS was added to the lower chamber to serve as a chemoattractant. After 4 h incubation, the solution in the lower chamber was collected. Transmigrated THP-1 cells remaining on the lower surface of the Transwell^®^ filter were dislodged by 5 min incubation with 5 mM EDTA. Dislodged THP-1 cells were combined with the solution collected from the lower chamber and centrifuged. Pelleted THP-1 cells were resuspended in 100 μL of RMPI 1640 medium supplemented with 10% FBS and measured in a plate reader. RFU were converted to the number of cells with a standard curve (not shown).

### Animal Studies

*Lrg1*^–/–^ mice on a C57BL/6N background were originally generated by the knockout mouse project repository^1^ and were a generous gift of Professor J. Greenwood, UCL Institute of Ophthalmology. Mouse colonies were housed in animal biosafety level 2 holding rooms and subjected to a 12 h light/dark cycle with humidity regulated at 55%. Mice had *ad libitum* access to standard chow diet and drinking water. Experimental mice were generated from random pairs of homozygous breeders. 37-week-old male *Lrg*^–/–^ and corresponding wild–type control mice were euthanised by cervical dislocation under anesthesia after exposure to 5% inhalant isoflurane (Piramal Healthcare, India) for 5 min. Blood was collected via cardiac puncture. Blood samples were stored in heparin-coated capillary tubes sealed with Critoseal^®^ (Leica Biosystems, Germany) at room temperature for 30 min for coagulation to occur. Plasma was obtained by centrifugation at 1600 × *g* for 10 min and stored at −80°C until further analysis. Animal experiments were performed in compliance with the guidelines of the Institutional Animal Care and Use Committee (ARF-SBS/NIE-A0251) of Nanyang Technological University, Singapore and the Guide for Care and Use of Laboratory Animals published by United States National Institutes of Health.

### Statistical Analysis

Data are presented as mean ± standard error of the mean. Statistical analyses were performed by Student’s *t*-test or one-way ANOVA with Bonferroni’s *post hoc* test using GraphPad Prism 6 (GraphPad Software Inc., United States). The criterion for significance was *p* < 0.05 (^∗^*p* < 0.05; ^∗∗^*p* < 0.01; ^∗∗∗^*p* < 0.001; and ^****^p < 0.0001).

## Results

### LRG1 Was Expressed in Activated Endothelium of Stenotic Arteries

**FIGURE 1:**
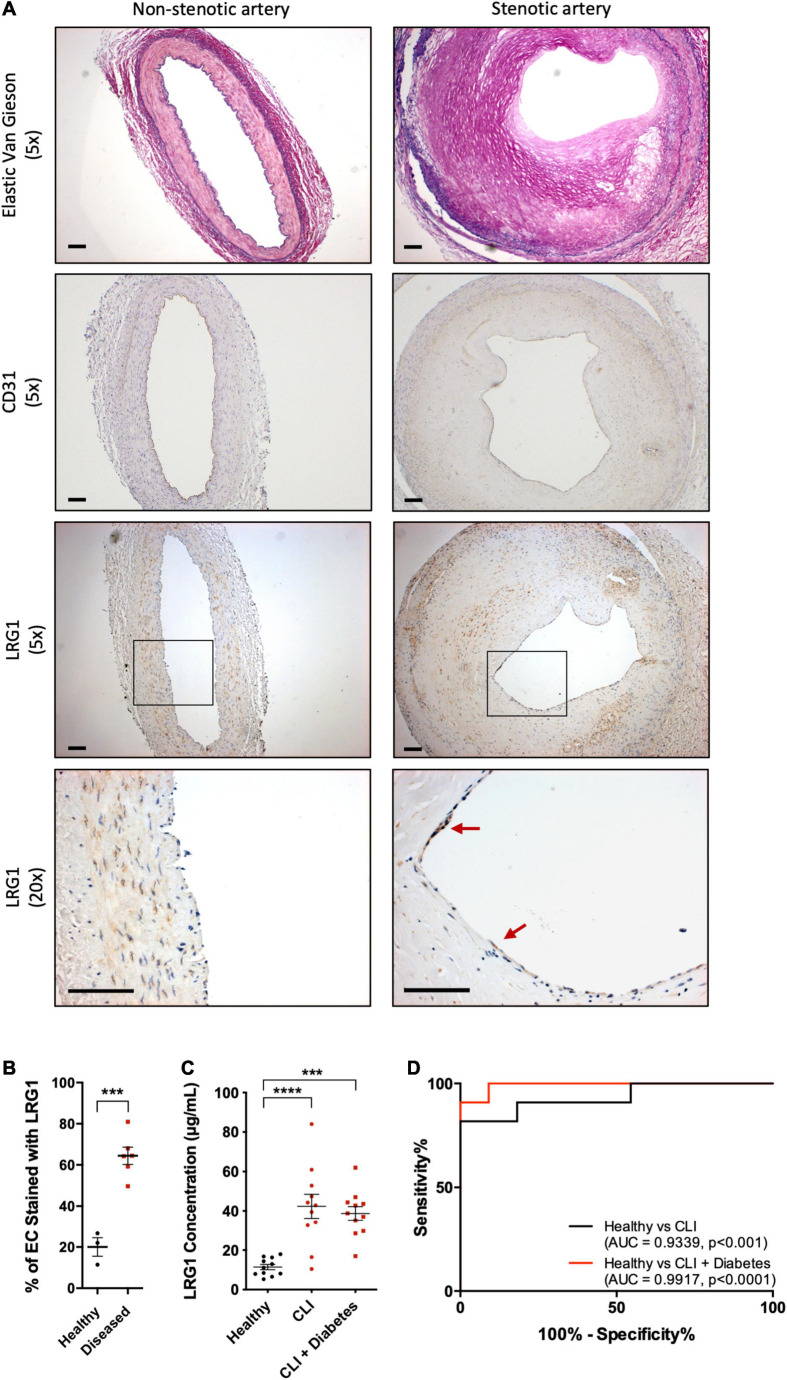
Leucine -rich α-2-glycoprotein (LRG1) was upregulated in patients with vascular disease. **(A)** Elastic Van Gieson (EVG) (first row) staining and immunohistochemical CD31 (second row) and LRG1 (third and fourth row) in sections of non-stenotic (left column) and stenotic (right column) coronary artery. LRG1 was highly expressed in endothelial cells of stenotic arteries compared to non-stenotic arteries. Red arrowheads show examples of LRG1-expressing endothelial cells. Scale bar = 100 μm. **(B)** The percentage of endothelial cells staining for LRG1 was significantly higher in diseased coronary arteries than non-diseased coronary arteries. **(C)** ELISA measurement of LRG1 concentration in plasma from healthy controls and critical limb ischemia (CLI) patients, with and without diabetes. LRG1 was significantly increased in the serum of CLI patients with and without diabetes. **(D)** Receiver Operating Characteristic (ROC) curve analysis for the ability of serum LRG1 to differentiate between healthy controls and CLI patients, and between healthy controls and CLI patients with diabetes. [**(B)**, Unpaired two-tailed Student’s *t*-test; *n* ≥ 3. **(C)**, One-way ANOVA followed by Bonferroni *post hoc* test; *n* = 11/group. ***p < 0.001; ****p < 0.0001].

To understand the association of LRG1 and atherosclerosis, paraffin-embedded sections of non-stenotic (*n* = 3) and stenotic (*n* = 6) coronary artery were stained with Elastic van Gieson stain (EVG) and anti-CD31 and anti-LRG1 antibodies (Figure 1A). The percentage of LRG1-expressing ECs in stenotic arteries was 3-fold higher than that in non-stenotic controls (Figure 1B, *p* < 0.001).

### LRG1 Concentration Was Higher in the Serum of CLI Patients

Atherosclerosis is the major cause of CLI. Consistent with the previous observation, LRG1 concentrations in the serum of CLI patients and diabetic CLI patients were significantly higher than those in healthy controls [42.34 ± 6.09 and 38.73 ± 3.51 versus 11.44 ± 1.35 μg/mL, *p* < 0.001 and *p* < 0.0001, respectively]. There was no significant difference in the concentration of LRG1 between the two groups of CLI patients (Figure 1C). The Area Under the Receiver Operating Characteristic (AUROC) yielded results of 0.9339 and 0.9917 for CLI patients and diabetic CLI patients, respectively (Figure 1D, *p* < 0.001 and *p* < 0.0001, respectively), demonstrating the ability of serum LRG1 concentration to differentiate CLI patients from healthy controls.

Multiple linear regression analysis was also performed and found that CLI was the only significant predictor of increased LRG1 concentration (Supplementary Table 1B, *p* < 0.01). Other factors (gender, diabetic condition, and age) did not contribute to the changes in LRG1 concentration.

### LRG1 Expression in ECs Was Induced by Atherogenic Flow and TNF-α

**FIGURE 2:**
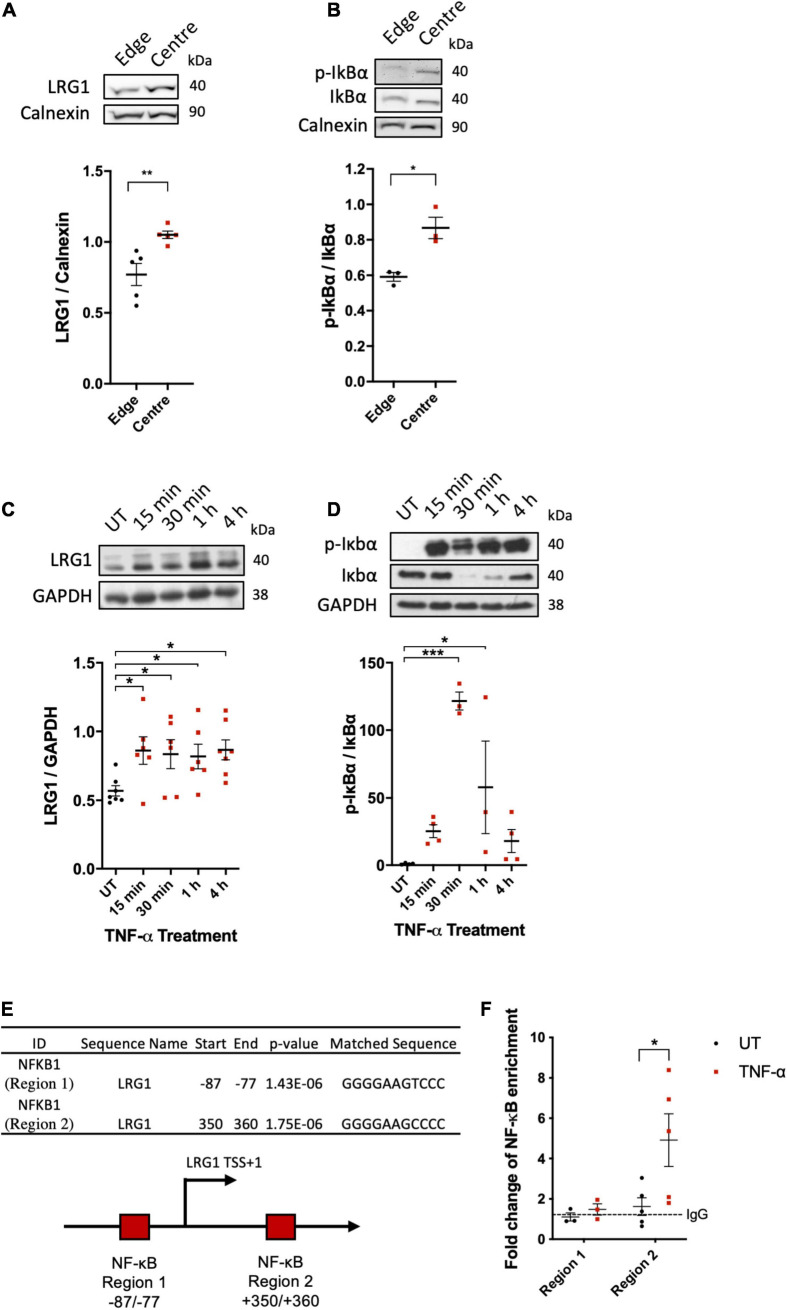
Leucine-rich α-2-glycoprotein (LRG1) protein expression were upregulated in activated EC. Putatively atherogenic flow at the centre of swirled wells upregulated the expression of **(A)** LRG1 and **(B)** phospho-IkBα in HUVECs, compared to putatively atheroprotective flow at the edge of the wells. Expression of **(C)** LRG1 and **(D)** phospho-IkBα was upregulated in HUVECs by TNF-α. **(E)** Summary of the motif analysis conducted using FIMO. The schematic diagram shows the 2 kb sequence near the transcription start site (bent arrow) of the LRG1 promoter region containing two putative NF-κB (RelA) binding sites (red box) predicted by FIMO. **(F)** ChIP-qPCR analysis of TNF-α-induced association between NF-κB/p65 and the LRG1 promoter in HDMECs. [**(A,B,E)**, Unpaired two-tailed Student’s *t*-test; *n* ≥ 3. **(C)**, One-way ANOVA followed by Bonferroni *post hoc* test; *n* ≥ 3. *p < 0.05; **p < 0.01].

Putatively atherogenic LMMF significantly upregulated HUVEC LRG1 protein levels and induced IκBα phosphorylation compared to putatively atheroprotective HMUF (Figures 2A,B, *p* < 0.01, *p* < 0.05, respectively). LRG1 protein levels and IκBα phosphorylation in HUVECs were also upregulated by TNF-α treatment (Figures 2C,D, *p* < 0.05, *p* < 0.05).

Consistent with the upregulation of LRG1 in activated ECs, bioinformatic analysis of the 2 kb sequence near the TSS of the LRG1 promoter region led to the identification of two potential NF-κB binding sites (Figure 2E). ChIP analysis showed binding of NF-κB to region 2 (downstream of TSS), but not region 1 (upstream of TSS), of the LRG1 promoter region of TNF-α-treated HDMECs (Figure 2F). These data suggest that LRG1 is a direct target gene of NF-κB.

### Effects of LRG1 on Monocyte Recruitment

**FIGURE 3:**
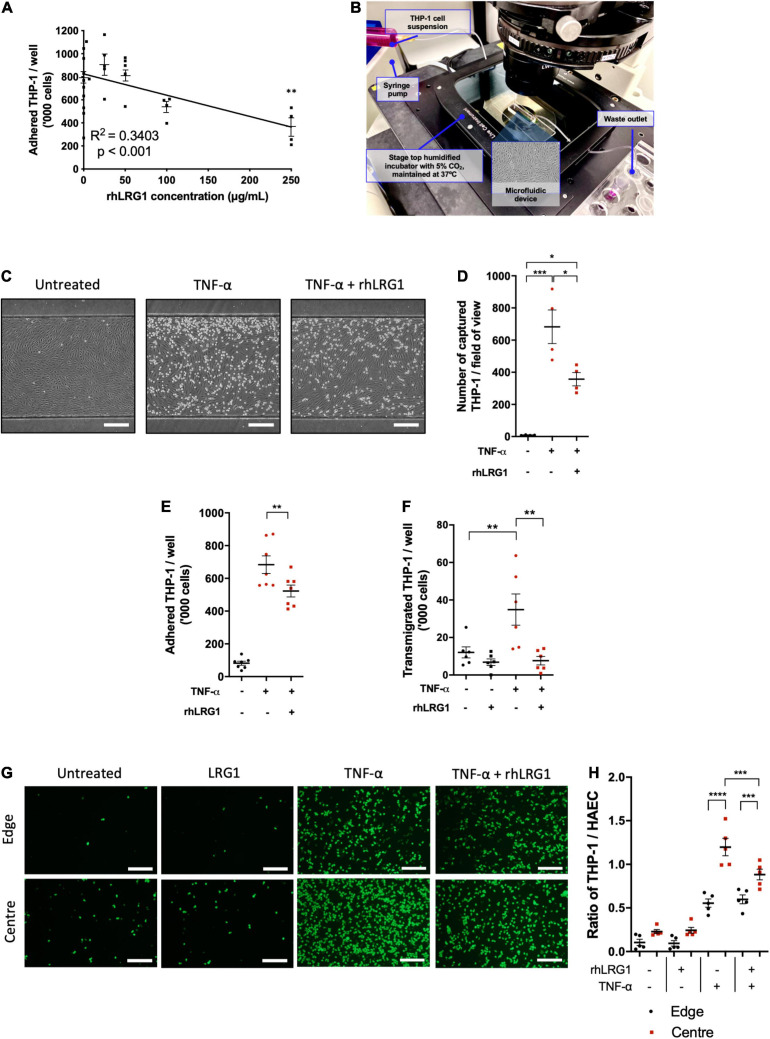
Recombinant human LRG1 (rhLRG1) suppressed THP-1 recruitment on TNF-α-activated EC. **(A)** rhLRG1 treatment suppressed THP-1 adhesion on HUVECs activated by TNF-α in a dose-dependent manner. Additional statistical testing compared each dose with non-treated cells, ns = not significant. **(B)** Setup of the THP-1 capture assay using a microfluidic chip. **(C)** Representative images of THP-1 captured on HUVECs under flow in a microfluidic chip. Scale bar = 100 μm. **(D)** rhLRG1 treatment prevented TNF-α-induced THP-1 capture on HUVECs under laminar flow in a microfluidic chip. **(E)** rhLRG1 treatment decreased TNF-α-induced THP-1 adhesion to HAECs. **(F)** rhLRG1 treatment prevented TNF-α-induced THP-1 migration across HAEC monolayers. **(G)** Representative images of Calcein-AM-stained THP-1 adhered to HAECs with different treatment and shear conditions. Scale bar = 200 μm. **(H)** THP-1 adhesion to HAECs sheared using a swirling well plate. rhLRG1 significantly suppressed TNF-α-induced THP-1 adhesion under putatively atherogenic flow. (One-way ANOVA followed by Bonferroni *post hoc* test; *n* ≥ 4. *p < 0.05; **p < 0.01; ***p < 0.001; ****p < 0.0001).

Recombinant human LRG1 had a dose-dependent inhibitory effect on TNF-α–induced THP-1 adhesion to HUVECs (Figure 3A, *R*^2^ = 0.3403, *p* < 0.001). The strongest inhibitory effect was observed with 250 μg/mL of rhLRG1, leading to a 53% reduction compared to HUVECs treated with TNF-α alone (Figure 3A, *p* < 0.01). For subsequent assays, 250 μg/mL of rhLRG1 was used.

There are a several stages in monocyte recruitment into vascular tissue, namely monocyte capture, firm adhesion, and transendothelial migration (Čejková et al., 2016). Different *in vitro* assays were used to study these processes *in vitro*. Compared to PBS-treated control cells, rhLRG1 suppressed THP-1 capture on TNF-α-activated HUVECs by 48% (Figures 3C,D, *p* < 0.05).

Recombinant human LRG1 treatment suppressed THP-1 adhesion to HAECs under static conditions by 24% (Figure 3E, *p* < 0.01).

Recombinant human LRG1 had no impact on baseline monocyte transendothelial migration but it significantly attenuated TNF-α-induced transendothelial migration of THP1 (Figure 3F, *p* < 0.01).

Concerning the interaction between flow and TNF-α, more THP-1 cells adhered to HAECs subjected to LMMF than HMUF under basal conditions and in the presence of TNF-α (Figures 3G,H, *p* < 0.0001). rhLRG1 had no effect on adhesion when TNF-α was combined with HMUF but it reduced adhesion when TNF-α was combined with LMMF flow. (It did not completely eliminate the difference between the HMUF and LMMF conditions) (Figures 3G,H, *p* < 0.001).

### LRG1 Suppressed TNF-α-Induced Endothelial Activation and Apoptosis

**FIGURE 4:**
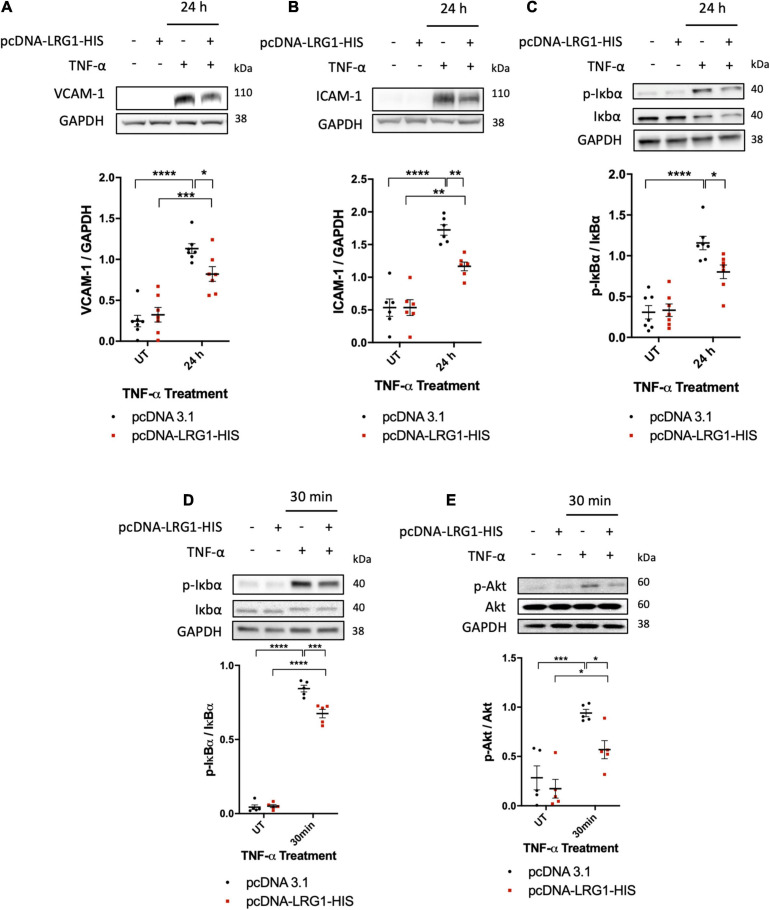
Leucine-rich α-2-glycoprotein (LRG1) overexpression suppressed activation by TNF-α. Representative blots and graphs of quantified **(A)** VCAM-1, **(B)** ICAM-1, and **(C)** IκBα in transfected cells. LRG1 overexpression in HUVECs suppressed VCAM-1, ICAM-1, and IκBα phosphorylation 24 h after TNF-α administration and suppressed **(D)** IκBα and **(E)** Akt phosphorylation 30 min after TNF-α administration, compared to HUVECs transfected with control plasmid. (One-way ANOVA followed by Bonferroni *post hoc* test; *n* ≥ 5. *p < 0.05; **p < 0.01; ***p < 0.001; ****p < 0.0001).

To investigate the effect of LRG1 on endothelial activation and TNF-α signaling, it was overexpressed in HUVECs. One day after transfection, Western blot analysis demonstrated elevated expression of LRG1 in the conditioned medium and in cell lysate (Supplementary Figure 1A). Transfected and control HUVECs were treated with TNF-α for 24 h to investigate the effect of LRG1 on VCAM-1 and ICAM-1 expression, both of which are downstream targets of NF-κB activation. There was significant suppression of TNF-α-induced expression of VCAM-1 and ICAM-1, and IκBα phosphorylation, in LRG1-overexpressing HUVECs (Figures 4A–C, *p* < 0.05, *p* < 0.01, *p* < 0.05, respectively).

Transfected and control HUVECs were also treated with TNF-α for 30 min to investigate the effect of LRG1 on downstream signaling. Thirty min was used because TNF-α-induced IκBα activation was found to peak at this time point (Figure 2D). At 30 min after TNF-α treatment, both IκBα phosphorylation (Figure 4D, *p* < 0.001) and Akt phosphorylation (Figure 4E, *p* < 0.05) were significantly suppressed in LRG1-overexpressing HUVECs compared to pcDNA3.1 transfected control cells.

Tumor necrosis factor-α-induced apoptosis and cleaved-caspase three levels were also suppressed (Supplementary Figure 2).

### LRG1 Desensitized TNF-α-Induced EC Activation by Inducing TNFR1 Shedding via ALK5 and ADAM10

**FIGURE 5:**
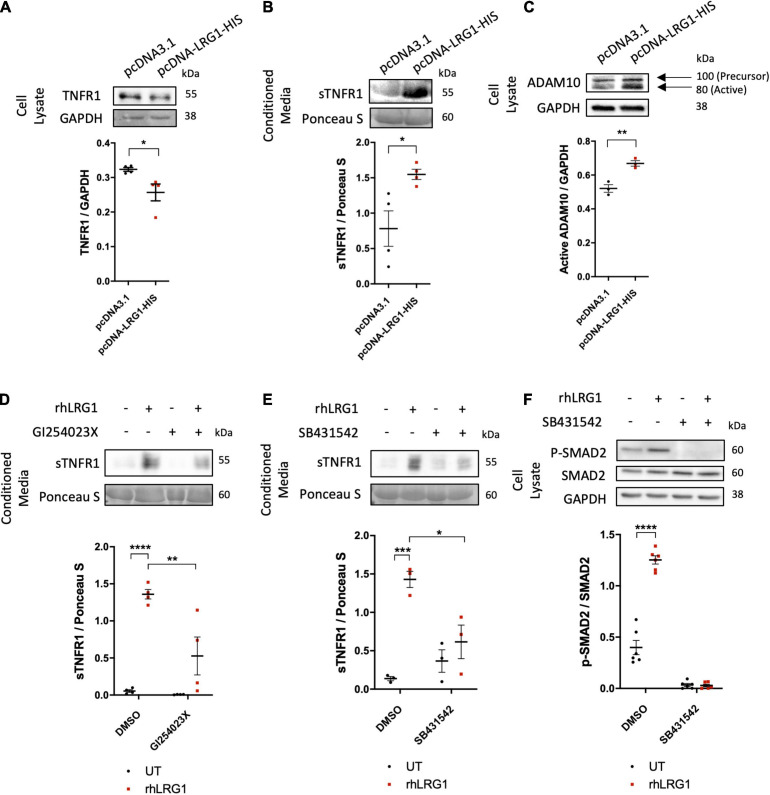
Recombinant human LRG1 (rhLRG1) induced TNFR1 shedding via ADAM10 and ALK5. Effect of LRG1 overexpression on levels of **(A)** TNFR1 and **(C)** ADAM10 in HUVECs, and **(B)** soluble TNFR1 (sTNFR1) in HUVEC-conditioned medium. **(D)** Levels of sTNFR1 in medium conditioned by HAECs that had been treated with rhLRG1 (with PBS as a control) and/or an ADAM10 inhibitor. rhLRG1 significantly increased the expression of sTNFR1 in conditioned medium and its activity was significantly reduced in the presence of an ADAM10 inhibitor. **(E)** sTNFR1 in the conditioned medium of HAECs and **(F)** SMAD2 in HAECs treated with rhLRG1 (with PBS as control) and/or an ALK5 inhibitor. rhLRG1 significantly increased the expression of sTNFR1 in conditioned medium and its activity was significantly reduced in the presence of the ALK5 inhibitor. rhLRG1 also increased SMAD2 activation, and the diminished pSMAD2 expression in HAECs validated the effect of the ALK5 inhibitor on HAECs. [**(A–C)**, Unpaired two-tailed Student’s *t*-test; *n* ≥ 3. **(D–E)**, One-way ANOVA followed by Bonferroni *post hoc* test; *n* ≥ 3. *p < 0.05; **p < 0.01; ***p < 0.001; ****p < 0.0001].

The mechanisms by which LRG1 overexpression suppressed effects of TNF-α were investigated. There was lower expression of TNF-α receptor 1 (TNFR1) in HUVECs overexpressing LRG1 and a higher level of soluble TNFR1 (sTNFR1) in conditioned medium than observed with pCDNA3.1-transfected control HUVECs (Figures 5A,B, *p* < 0.05, *p* < 0.05).

The active form of ADAM10 protein was upregulated in HUVECs transfected with pcDNA-LRG1-HIS plasmid compared to HUVECs transfected with control plasmid (Figure 5C, *p* < 0.01), whereas ADAM17 expression was not changed (Supplementary Figure 3A). GI254023X, a potent ADAM10 inhibitor, significantly attenuated the rhLRG1-induced increase of sTNFR1 in HAEC-conditioned medium (Figure 5D, *p* < 0.01).

SB431542 (an ALK5 inhibitor), but not LDN193189 (an ALK1 inhibitor), suppressed the rhLRG1-induced shedding of TNFR1 (Figure 5E and Supplementary Figure 3B, *p* < 0.05, *p* > 0.05) and also an rhLRG1-induced increase in SMAD2 phosphorylation in HAECs (Figure 5F; *p* < 0.0001).

Collectively, these results suggest that LRG1 attenuates TNFα-mediated endothelial cell activation by inducing TNFR1 shedding in an ALK5- and ADAM10-dependent manner.

### Serum Concentration of sTNFR1 Correlated With LRG1 in Human Subjects and *Lrg1*^–/–^ Mice

**FIGURE 6:**
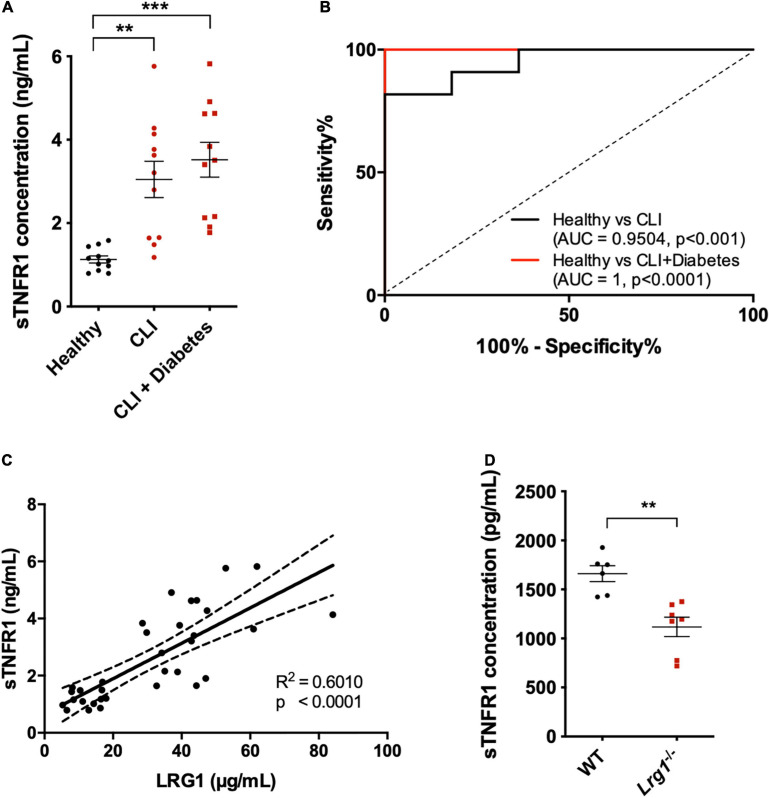
Soluble TNFR1 (sTNFR1) was upregulated in the serum of CLI patients and was correlated with LRG1 expression. **(A)** ELISA measurement of sTNFR1 concentration in healthy controls and CLI patients (with and without diabetes). sTNFR1 was significantly higher in the serum of CLI patients (with and without diabetes) compared to healthy controls. **(B)** Receiver Operating Characteristic (ROC) curve analysis of the ability of serum sTNFR1 to differentiate between healthy controls and CLI patients, and between healthy controls and CLI patients with diabetes. **(C)** The concentrations of sTNFR1 and LRG1 in healthy controls and CLI patients were significantly correlated. **(D)** ELISA measurement revealed that the concentration of sTNFR1 in the serum of 37-week-old *Lrg1^–/–^* mice was significantly lower than that in the serum of wild-type counterparts. [**(A)**, One-way ANOVA followed by Bonferroni *post hoc* test; *n* = 11/group. **(C)**, Linear regression analysis, *n* = 33; Dashed lines indicate the 95% confidence intervals for the regression line. **(D)**, Unpaired two-tailed Student’s *t*-test; *n* ≥ 6. **p < 0.01; ***p < 0.001].

Further confirmation of the role of sTNFR1 was conducted *in vivo*. sTNFR1 concentrations were higher in the serum of CLI patients, irrespective of the presence of diabetes, than in the serum of healthy controls [3.048 ± 0.436 and 3.519 ± 0.419 versus 1.128 ± 0.084 μg/mL, *p* < 0.01 and *p* < 0.001, respectively (Figure 6A)]. AUROC analysis yielded results of 0.9504 and 1 for CLI patients and diabetic CLI patients, respectively (Figure 6B, *p* < 0.001 and *p* < 0.0001, respectively), demonstrating the ability of sTNFR1 concentrations to differentiate CLI patients from healthy controls. Furthermore, there was a positive correlation between the expression of LRG1 and sTNFR1 in human serum (Figure 6C, *R*^2^ = 0.601, *p* < 0.0001).

Thirty-seven-week-old *Lrg1*^–/–^ mice (*n* = 6) expressed 33% lower sTNFR1 concentration than wild-type mice (*n* = 7, Figure 6D, *p* < 0.01).

## Discussion

Elevated serum LRG1 levels are associated with various diseases, including different types of cancer (Andersen et al., 2010; Furukawa et al., 2015; Zhang et al., 2018), inflammatory bowel disease (Serada et al., 2012), joint disorders (Ha et al., 2014), and cardiovascular diseases (Watson et al., 2011; Yin et al., 2014; Bos et al., 2017; Xie et al., 2019). Recently, Pek et al. (2015) have demonstrated that type 2 diabetes (T2D) patients with peripheral artery disease (PAD) had higher plasma LRG1 concentrations than T2D patients without PAD. Consistent with this observation, we observed significantly higher serum LRG1 in patients with CLI, an advanced stage of PAD that results from a progressive narrowing or blockage of limb arteries as a consequence of atherosclerosis (Santilli and Santilli, 1999). There was no difference in serum LRG1 levels between CLI patients with diabetes and without diabetes, suggesting that the increase in circulating LRG1 is specifically associated with CLI. Outcome data were not available for this cohort, but it would be interesting to study the association of plasma LRG1 levels with patient outcomes in CLI.

To further explore the association between LRG1 and atherosclerosis, we investigated the LRG1 expression pattern in coronary artery samples. LRG1 expression was higher in the endothelium of stenotic coronary arteries compared to non-stenotic coronary arteries, suggesting a potential role for LRG1 in endothelial homeostasis and arterial disease. This finding is consistent with the elevated concentration of serum LRG1 in CLI patients. However, it is not clear whether the up-regulated expression of LRG1 was the cause or the consequence of endothelial dysfunction.

To further understand this, we investigate the role of LRG1 in endothelial activation. ECs were exposed to two well-established inducers of endothelial activation, namely atherogenic flow (Ghim et al., 2017) and TNF-α (Read et al., 1994). Although it has previously been reported that shear stress regulates LRG1 gene expression, only normal versus pathologically elevated uniaxial shear stress, relevant to arteries that are already stenotic, was investigated (GEO DataSet GDS3868) (White et al., 2011). LMMF, on the other hand, is implicated in the initiation of atherosclerotic lesions (Peiffer et al., 2013). Hence, we applied LMMF (putatively atherogenic flow) and HMUF (putatively atheroprotective flow) using the swirling-well system (Ghim et al., 2018) and demonstrated that LRG1 expression and NF-κB activation were significantly higher in the ECs exposed to putatively atherogenic flow.

Consistent with the previous observation by Wang et al. (2017), our study showed that TNF-α treatment also upregulated LRG1 expression in HUVECs. Interestingly, TNF-α induced a prompt increase in LRG1 protein levels. LRG1, a secreted glycoprotein, has been reported to be packaged into the granule compartment of human neutrophils and secreted upon neutrophil activation (Druhan et al., 2017). LRG1 might similarly be stored in secretory vesicles of ECs and released quickly upon activation by external stimuli. Further studies are required to establish the mechanism of TNF-α-mediated LRG1 secretion in ECs.

It was also observed that NF-κB activation peaked at 30 min of TNF-α treatment (Figure 2D). Although this was followed by a sharp decline, activation had not returned to its basal level by 4 h. Figure 4C shows that IκBα phosphorylation could be seen in HUVECs 24 h following TNF-α treatment. These results are consistent with the work of Hoffmann et al. (2002) who showed that NF-κB activation exhibits oscillatory behavior, peaking at 30 min of TNF-α stimulation before being damped and maintained above baseline activation. A sustained increase of LRG1 expression in TNF-α-treated HUVECs is consistent with this temporal pattern of NF-kB activation.

Since both LMMF and TNF-α can induce NF-κB activation, we investigated the possibility of NF-κB-dependent induction of LRG1 expression by bioinformatics analysis and identified two potential NF-κB binding sites within the LRG1 promoter region. ChIP assay further validated the transcriptional control of LRG1 by the binding of NF-κB to its promoter region located downstream of TSS.

The evidence that NF-κB activation lies upstream of LRG1 may explain why LRG1 expression is sensitive to flow: NF-κB is activated by application of shear. A mechanosensory complex involving platelet endothelial cell adhesion molecule (PECAM-1), vascular endothelial cell cadherin (VE-cadherin), and vascular endothelial growth factor receptor 2 (VEGFR2) is sufficient to confer this behavior (Tzima et al., 2005). The shear stress profiles applied in the present study were complex and mechanosensors tend to act in concert, so other pathways may also be involved.

Activated ECs express high levels of cell adhesion molecules such as VCAM-1 and ICAM-1 that mediate monocyte capture, adhesion and subsequent transendothelial migration (Gerhardt and Ley, 2015). These steps are critical in the initiation of atherogenesis (Mestas and Ley, 2008). For instance, mice with mutated VCAM-1 and ICAM-1 have decreased atherosclerosis compared to control mice (Bourdillon et al., 2000; Cybulsky et al., 2001). To understand the functional consequence of elevated LRG1 expression in EC activation, we transfected ECs with a LRG1-expressing plasmid or subjected ECs to treatment with rhLRG1. LRG1 reduced IκBα and Akt phosphorylation after 30 min of TNF-α treatment and reduced the expression of VCAM-1 and ICAM-1 (both of which are downstream of the NF-κB pathway).

We further showed that LRG1 reduced not only adhesion molecule expression but also monocyte capture on ECs under flow conditions, adhesion to ECs under static and flow conditions, and transendothelial migration. These findings imply that LRG1 upregulation in human disease could prevent monocyte-endothelial interactions and monocyte infiltration into the sub-endothelial space. To the best of our knowledge, this is the first study showing that LRG1 has a regulatory role in EC activation and endothelial-monocyte interactions.

Tumor necrosis factor-α is known to activate the NF-κB pathway by binding to TNFR1 (Fischer et al., 2015). Akt phosphorylation and the activation of caspase-3-dependent apoptotic pathway are also mediated by this receptor (Sedger and McDermott, 2014). TNFR1 shedding has been shown to diminish TNF-α-induced endothelial activation and it is an important process in immunomodulation (Sommer et al., 2017). Knock-in mice with mutated non-cleavable TNFR1 are more susceptible to inflammation and autoimmune disorders (Xanthoulea et al., 2004). Mutations have been found in the extracellular domain of TNFR1 in patients with autoinflammatory syndromes (McDermott et al., 1999). Shedding of TNFR1 from cell membrane desensitizes cells to TNF-α ligands, and soluble TNFR1 acts as an antagonist to circulating TNF-α (Giai et al., 2013).

We therefore tested whether LRG1 exerts its function by interfering with the activity of TNFR1. We showed that overexpression of LRG1 in HUVECs resulted in the shedding of the TNFR1 ectodomain into conditioned medium, suggesting that the inhibitory effect of LRG1 on TNF-α-induced EC activation is mediated by post-translational modification of TNFR1. Structural analysis of the LRG1 protein sequence revealed an absence of known protease domains, but we showed that LRG1 increased the level of the active form of ADAM10, a sheddase with the known role of releasing soluble TNFR1 ectodomain in other contexts (Yang et al., 2015, 2017). Consistent with this observation, the effect of LRG1 on TNFR1 shedding in HUVECs was partially abrogated in the presence of an ADAM10 inhibitor. No change was observed in the expression of ADAM17, another enzyme commonly involved in TNFR1 shedding. Note that LRG1-dependent shedding of other ADAM10 substrates was not investigated in this study; future studies are warranted to more comprehensively investigate the role of the LRG1-ADAM10 axis in ECs.

LRG1 has a strong pro-angiogenic effect that is mediated by promoting TGFβ-induced activation of the Smad1/5/8 pathway through the angiogenic TGFβ type I receptor, ALK1, in the presence of endoglin (Wang et al., 2013b). Although LRG1 is able to bind directly to the angiostatic TGFβ type I receptor, ALK5, its impact on TGFβ-induced activation of SMAD2/3 signaling is negligible in HUVECs (Wang et al., 2013b). The present study showed that LRG1 alone is able to activate SMAD2 signaling in HAECs. To further understand the involvement of different TGFβ receptors in LRG1-mediated TNFR1 shedding, HUVECs were treated with specific ALK1 and ALK5 inhibitors. Inhibition of ALK5, but not ALK1, partially attenuated the ability of LRG1 to release the TNFR1 ectodomain. The fact that the LRG1-mediated TNFR1 shedding was not fully abrogated by ALK5 inhibition implies the presence of other mechanisms.

The association between TNFR1 shedding and LRG1 was also investigated in serum from human CLI patients and *Lrg1*^–/–^ mice. The concentration of sTNFR1 was previously reported to depend, at least in part, on TNFR1 shedding (Hawari et al., 2004). Our study showed that CLI patients had significantly higher serum concentrations of soluble sTNFR1, which is consistent with other studies of coronary atherosclerosis, and of arthritis (Kim et al., 2016). We further showed a positive correlation between the concentration of LRG1 and sTNFR1 in the serum. Also consistent with a critical role for LRG1 in TNFR1 shedding, there was a reduced serum level of sTNFR1 in *Lrg1*^–/–^ mice compared to wild type mice. Note that circulating sTNFR1 was not completely eliminated in *Lrg1*^–/–^ mice, however, suggesting the existence of other pathways for TNFR1 shedding.

It is worth noting that this study does not attempt to establish the role of LRG1 and ALK5-SMAD2/ADAM10-dependent TNFR1 shedding in CLI. Although we have shown that serum LRG1 and sTNFR1 concentrations are positively correlated in CLI patients, it is not currently possible to exclude an independent impact of chronic inflammation on both LRG1 and sTNFR1 levels. It is also challenging to provide direct experimental evidence for a role of LRG1 and ALK5-SMAD2/ADAM10-dependent TNFR1 shedding in CLI *in vivo* because the CLI mouse model has a different disease etiology from that in human patients and knockout of ADAM10 in mice results in embryonic lethality (Jorissen et al., 2010).

Since LRG1 is able to cause the shedding of TNFR1 and its expression is shear dependent, it is possible that the shedding of TNFR1 is also dependent on the shear profile. An effect of this type has recently been reported in osteoblasts, where oscillatory shear stress reduced the amount of TNFR1 on the cell surface (Wang et al., 2011).

The NF-κB pathway has long been proposed as a therapeutic target in inflammatory and autoimmune diseases, but complete inhibition of TNF-α using neutralizing reagents has resulted in disease exacerbation, risk of infection, and other side effects related to immune regulation and tissue regeneration (reviewed by Fischer et al., 2015). Attention has therefore been redirected to the modulation of TNFR1. In light of the role of LRG1 in TNFR1 shedding, the potential therapeutic role of LRG1 in inflammatory and autoimmune diseases, including in atherosclerosis, should be investigated further.

**FIGURE 7:**
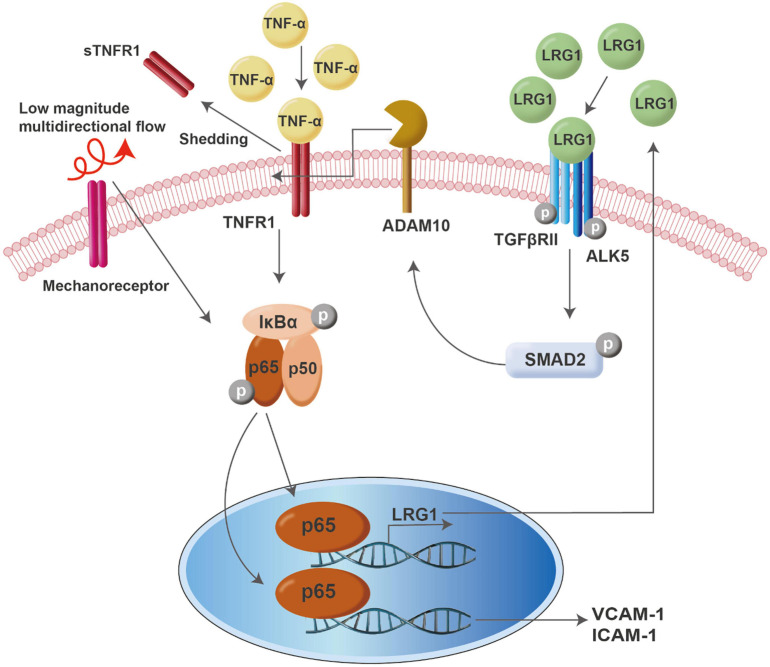
Model of LRG1 activity in endothelial activation.

In conclusion, our findings suggest that LRG1 expression is triggered by pro-inflammatory cytokines and atherogenic flow through transcriptional activity of NF-κB and may serve as a negative regulator of inflammation. Initiation of an inflammatory event is generally also accompanied by activation of pathways that inhibit the inflammation, to prevent a runaway cascade. An example is that nuclear translocation of NF-κB increases transcription of IκB. Here we speculate, similarly, that pro-inflammatory mechanical or cytokine stimulation releases LRG1, which has anti-inflammatory actions. LRG1 exerts its function by the shedding of TNFR1 via ALK5-SMAD2 signaling and ADAM10 activation. Upregulation of LRG1 expression in diseases like atherosclerosis might have an important role in modulating endothelial activation. Understanding LRG1 activity in endothelial activation might provide a new avenue for therapeutic discovery in inflammatory and autoimmune diseases. A model for the actions of LRG1 in endothelial activation is shown in Figure 7.

## Data Availability Statement

The original contributions presented in the study are included in the article/Supplementary Material, further inquiries can be directed to the corresponding author/s.

## Ethics Statement

The studies involving human participants were reviewed and approved by Hammersmith Hospital Research Ethics Committee. The patients/participants provided their written informed consent to participate in this study. The animal study was reviewed and approved by Institutional Animal Care and Use Committee of Nanyang Technological University, Singapore.

## Author Contributions

KP, PW, and XW conceived and designed the project and wrote the manuscript. KP, PS, AC, MY, FW, and RW acquired, processed, and analyzed human clinical samples. KP, MG, CL, HT, HH, CF, and CW carried out *in vitro* experiments. KP and RC acquired, processed, and analyzed mice plasma samples. All authors discussed the results and approved the final manuscript.

## Conflict of Interest

The authors declare that the research was conducted in the absence of any commercial or financial relationships that could be construed as a potential conflict of interest.

## Footnotes

1 http://www.komp.org
