# Supplementary material for: Leucine-Rich α-2-Glycoprotein 1 Suppresses Endothelial Cell Activation Through ADAM10-Mediated Shedding of TNF-α Receptor
Source: Front Cell Dev Biol. 2021 Jul 5;9:706143. doi: 10.3389/fcell.2021.706143 (PMC8288075; doi:10.3389/fcell.2021.706143)
Supplement: Supplementary file 1 [file Data_Sheet_1.docx]

**Supplementary Material:**

**Supplementary Table**

**Supplementary Table 1**

(a) Clinical characteristics of critical limb ischemia (CLI) patients. (b) Multiple linear regression was performed using CLI, gender, diabetic condition, and age to determine the predictor of LRG1 concentration. Only CLI was found to be a significant predictor of increased LRG1 concentration. (Multiple linear regression; n=33. **p<0.01)

**Supplementary Table 2**

List of antibodies used for western blot.

**Supplementary Figure**


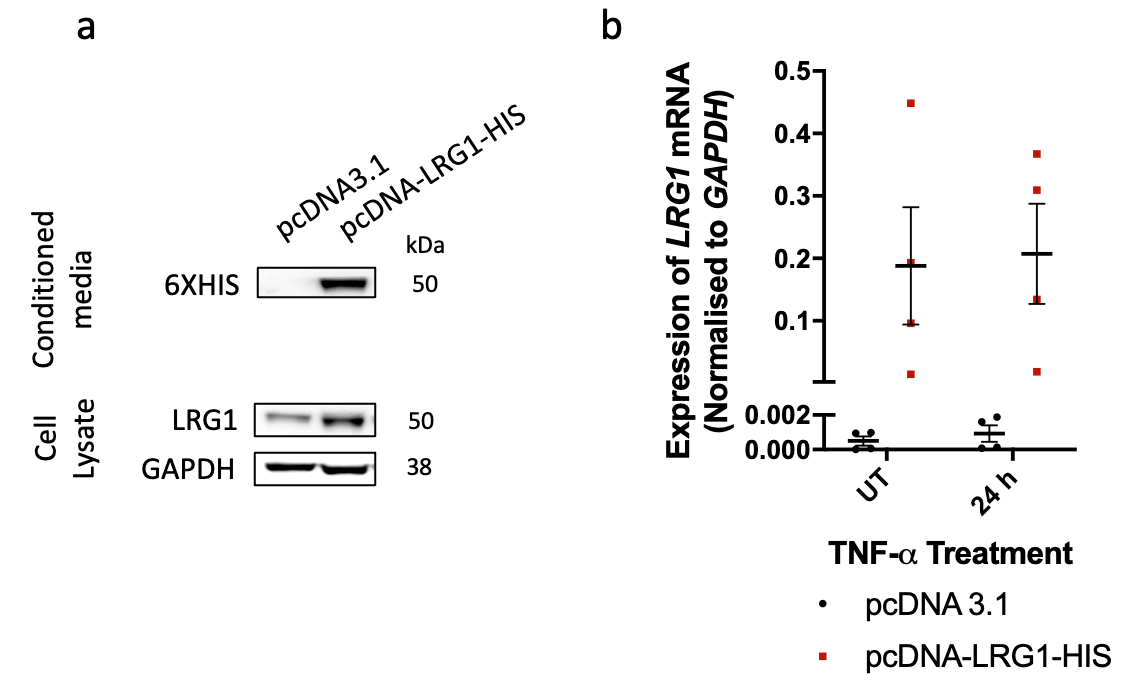


**Supplementary Figure 1**

(a) The presence of 6xHIS protein in conditioned medium and the increased protein and (b) mRNA expression of LRG1 in cell lysate show that cells were successfully transfected with pcDNA-LRG1-HIS plasmid.

**Supplementary Figure 2**

LRG1 overexpression significantly suppressed TNF-α-induced cell death compared to cells transfected with control plasmid. Number of (a) non-adhered, (b) adhered cells, (c) trypan blue-stained adhered cells, and (d) total number of cells grown in a 6-well plate transfected with control or LRG1 plasmid, with or without (“UT”) TNF-α treatment. (Total number of cells = Non-adhered cells + adhered cells). (e) Percentage of dead cells in transfected HUVECs with or without TNF-α treatment. Overexpression of LRG1 significantly suppressed TNF-α-induced cell death compared to cells transfected with control plasmid. (f) Representative Western blots of cleaved caspase 3 in transfected HUVECs with or without TNF-α treatment. (g-h) Densitometry analysis of cleaved caspase 3 (17kDa and 19kDa) in transfected HUVECs with or without TNF-α treatment. Overexpression of LRG1 significantly suppressed TNF-α-induced cleaved caspase 3 compared to cells transfected with control plasmid. (One-way ANOVA followed by Bonferroni post hoc test; n≥3. *p<0.05; **p<0.01; ****p<0.0001)


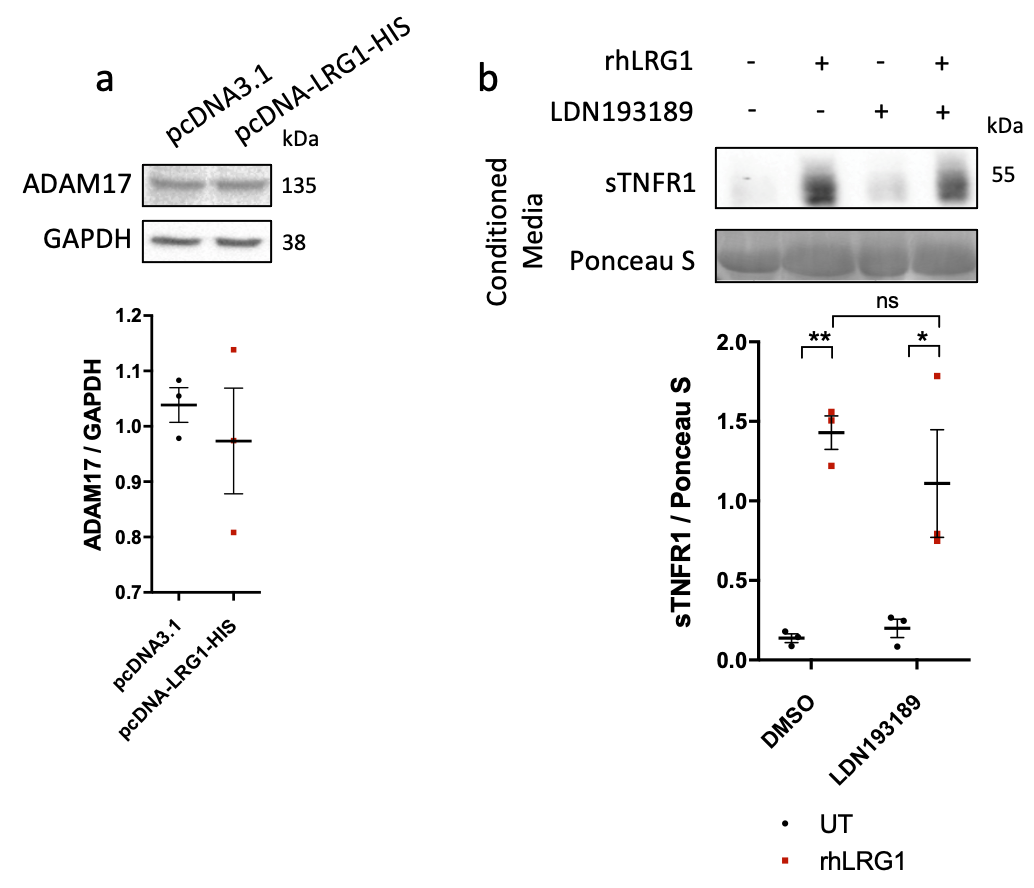


**Supplementary Figure 3**

(a) Representative Western blots and densitometric analysis of the expression of ADAM17 in transfected HUVECs. LRG1 overexpression had no effect on ADAM17 expression. (b) Representative Western blots and densitometric analysis of the level of sTNFR1 in the conditioned medium of HAECs treated with rLRG1 (with PBS as control) and/or an ALK1 inhibitor. rLRG1 significantly increased the expression of sTNFR1 in conditioned medium, and its activity was unaffected in the presence of the ALK1 inhibitor. (a, Unpaired two-tailed Student’s t-test. b, One-way ANOVA followed by Bonferroni post hoc test; n≥3. *p<0.05; **p<0.01)

**Supplementary Methods**

## Quantitative reverse transcription polymerase chain reaction (qRT-PCR)

Total RNA was extracted from ECs using RNAzol RT (Molecular Research Centre, USA) according to the manufacturer’s protocol. 1 μg of RNA was reverse transcribed into cDNA using qScript cDNA Supermix (Quanta Biosciences, USA). qRT-PCR was performed with PrecisionFAST qPCR MasterMix (PrimerDesign, UK) using the Applied Biosystems QuantStudio 6 Flex Real-Time PCR System (ThermoFisher Scientific, USA). Primer sequences are shown below:

LRG1, forward primer, 5’-GATCGTGCCACTGCACTCTA-3’, reverse primer, 5’- GAAAGCCCATCGTGTGTTCT-3’.

GAPDH, forward primer, 5’-GGTCTCCTCTGACTTCAACA-3’, reverse primer, 5’- AGCCAAATTCGTTGTCATAC-3’

## Trypan blue exclusion assay

The trypan blue (Sigma-Aldrich, UK) exclusion assay was used to determine the viability of transfected EC treated with TNF-α. Trypan blue solution was added to trypsinised EC which were then loaded on to a haemocytometer to count the blue stained cells and the total number of cells. Culture medium was centrifuged to harvest the floating cells and the cells were counted using a haemocytometer. The percentage of dead cells were calculated and presented to show the effect of treatment on cell viability. [Percentage of dead cells = (Non-adherent cells + stained adherent cells) / (non-adherent cells + adherent cells) x 100%]
